# Hepatitis B Virus Infection in Pregnancy: Immunological Response, Natural Course and Pregnancy Outcomes

**DOI:** 10.3390/jcm10132926

**Published:** 2021-06-29

**Authors:** Sirinart Sirilert, Theera Tongsong

**Affiliations:** Department of Obstetrics and Gynecology, Faculty of Medicine, Chiang Mai University, Chiang Mai 50200, Thailand; sirinart.s@cmu.ac.th

**Keywords:** hepatitis B virus, immunological response, outcomes, pregnancy

## Abstract

This review aimed to provide an update on the impact of pregnancy on the natural course of hepatitis B virus (HBV) infection and also on the impact of HBV infection on adverse pregnancy outcomes, including mother-to-child transmission (MTCT). For the literature review, original research articles, review articles, and guidelines were narratively reviewed and comprehensively validated. The databases of PubMed, EMBASE, and CINAHL were carefully searched for articles in English on topics related to HBV infection, pregnancy, and vertical transmission from 1960 to May 2021. Immunological changes during pregnancy such as suppression of Th1 response and induction of Th2 immunity lead to an impaired immune reaction to HBV and stimulate viral activity along with the reduction of CD8 T cells to escape immune detection. The impact of pregnancy on the natural course of chronic HBV infection seems to be minimal, while pregnancy can increase morbidity and mortality in the case of advanced HBV hepatitis or cirrhosis. Importantly, hepatitis flare or alanine aminotransferase (ALT) flare can occur during pregnancy and is more common during the postpartum period due to the interaction between HBV and the immune response. Interestingly, the impact of HBV infection on adverse pregnancy outcomes is more serious than ever thought. Updated evidence indicates that pregnancies with chronic HBV infection increase the risk of preterm birth and gestational diabetes, especially in cases of positive hepatitis e antigen (HBeAg).

## 1. Introduction

In the past decades, hepatitis B virus (HBV) infection has been studied extensively. However, the studies of the natural course of the infection during pregnancy are relatively limited, in spite of the fact that maternal-to-child transmission is the main transmission in many parts of the world. Moreover, although immunoprophylaxis to prevent vertical transmission has been widely used, vertical transmission is still a leading cause of high prevalence. In recent decades, immunoprophylaxis failure has been explored. It has been demonstrated that the number of viral copies is associated with prophylaxis failure, resulting in a big change in clinical practice, especially antiviral therapy to lower the transmission rate. The mechanism of transmission is less understood. It might not be as had ever been thought that the main infection is contamination during labor and delivery. Antenatal placental transmission may be a significant cause of failure of prophylaxis after birth. This review updates intrauterine transmission. Additionally, several studies suggest that HBV can increase adverse outcomes, such as intrahepatic cholestasis of pregnancy, gestational diabetes, and preterm birth. Such associations seem to be related to the activity of the HBV. Finally, pregnancy can modify the natural course of HBV infection, especially flare up of hepatitis. This review focuses on transplacental infection, natural course, and pregnancy outcomes, including mother-to-child transmission.

Chronic hepatitis B virus (HBV) infection affects more than 290 million people worldwide, causing a wide range of health problems, e.g., hepatitis, cirrhosis, hepatic failure, and hepatocellular carcinoma (HCC), leading to 887,000 deaths per year worldwide, in spite of the current availability of an HBV vaccine. The prevalence of HBV infection is categorized into three geographical areas: high (>8%: East Asia, Africa), medium (2–8%: Mediterranean, Eastern Europe), and low (<2%: North America, Western Europe) [1]. Over the past decade, the epidemiology has been changing because of universal vaccination, HBV screening programs, and the migration of people between low and high prevalence regions. Surprisingly, the current predominant route of HBV is mother-to-child transmission (MTCT), accounting for approximately 50% of the number of global patients [2]. In the high endemic regions, MTCT is the main route of infection, and the risk of progression to chronicity is high.

HBV infection is one of the global health issues. Viral hepatitis, especially HBV followed by hepatitis C virus (HCV), is the seventh leading cause of death. The World Health Organization (WHO) and Global Burden of Disease study demonstrated that the mortality rate of viral hepatitis has increased in comparison with the mortality rate of tuberculosis, and it is higher than that of human immunodeficiency virus (HIV) infection [3,4]. Deaths caused by HBV infection result from acute hepatitis in newly infected patients, progression of disease from asymptomatic carriers to acute symptomatic or hepatitis flare, slow progression to decompensated cirrhosis, and hepatocellular carcinoma (HCC) [5]. Around 20–30% of chronic carriers would develop HBV decompensated complications (such as cirrhosis, a condition characterized by alteration of liver function and architecture) or HCC, and most cases appear during adulthood after a long period of chronic HBV infection [4]. Additionally, chronic HBV infection is also involved with another mechanism of its viral pro-oncogenicity, which can directly induce the HBV infected hepatic cells to develop HCC, different from other causes of HCC that usually need a progression of the infected liver cells to be cirrhosis prior to development of HCC. [6].

In 1992, the WHO advocated HBV vaccination of all infants and children with three doses within the first year of life. In 2016, the WHO South-East Asia Regional Office (SEARO), covering 11 countries including 2 billion people, endorsed the control target of HBsAg seroprevalence of 1% or less, among children aged 5 year or more by 2020. Of the 11 countries, 4 could achieve the target [7]. In May 2016, the World Health Assembly endorsed the Global Health Sector Strategy (GHSS) on viral hepatitis 2016–2021. The GHSS calls for the elimination of viral hepatitis as a public health threat by 2030 (reducing new infections by 90% and mortality by 65%) [4]. Certainly, this target is unlikely to be achieved with the existing strategy. Currently, attention should focus on reducing HBsAg positivity to below 0.1% by the year 2030. To achieve the target, the capacity and quality of the health care system of individual regional areas must be critically analyzed. In addition to capacity of implementation of birth-dose vaccine and the coverage of three doses, prenatal care with assessment of viral loads and HBeAg, and the use of antiviral therapy in selected pregnant women should be strongly considered.

## 2. Immunological Effect of HBV Infection

### 2.1. Immune Response to HBV Infection during Pregnancy

A successful pregnancy needs immune adaptation to avoid fetal allograft rejection. The suppression of Th1 response and induction of Th2 immunity leads to an impaired immune reaction to HBV and stimulates viral activity along with the reduction of CD8 T cells to escape immune detection, thereby enhancing vertical transmission since CD8 T cells are the main effector cells responsible for T cell response [8]. Additionally, the pathogenesis during acute HBV infection and viral clearance is mediated by both noncytolytic and cytolytic effector functions of the CD8 T cells [8].

Other than normal immune adaptation during pregnancy, HBV-specific T cell epitopes and responses are important factors. The number of known HBV-specific T cell epitopes and their function are unclear. Moreover, there is limited evidence to support the occurrence of viral evolution during the development of chronic infection. A study by Desmond (2012) analyzed viral polymorphisms and their associations with the HLA types across the full HBV genome with high-resolution 4-digit HLA class I and II typing and full-length HBV sequencing; the study demonstrated 49 statistically significant associations between HLA types and HBV sequence variations at 41 sites in the HBV genome and found 78% HLA binding prediction [9]. Moreover, the study found a significant association between viral adaptation and the absence of HBeAg in a host, which further explained the concept of significant immune pressure on HBV virus before HBeAg seroconversion, causing a viral evolution that is called enhanced viral adaptation. However, no evidence of association between enhanced viral adaptation and HBV DNA levels or alanine aminotransferase (ALT) level was reported.

Contrary to the suppression of cell-mediated immunity to prevent rejection of the fetus, reversible immune response with high replication of HBV can occur during pregnancy and postpartum periods. The results of the flare are increased HBV replication, along with elevated levels of aminotransferases [10].

### 2.2. Fetal Immune Response

Fetal responses regarding the exposure of the immature fetal immune system to HBV were studied in HBeAg-positive mothers with suspected transfer of HBeAg through the placenta to the fetus. The exposure of the fetus to HBeAg could induce fetal T helper cell tolerance to HBeAg and HBcAg because of the cross reaction [11], increased regulatory T cells, and dysfunctional CD8 T cell. These immune responses were demonstrated in HBsAg-positive newborns with HBV DNA detected at birth; therefore, the fetus may develop immune-tolerance to HBV infection in utero [11,12,13]. Long term exposure to HBV and fetal immune-tolerance could be the causes of immunoprophylaxis failure or persistent infection. However, newborns have a very good response to neonatal vaccination. This may be due to the fact that the main cause of MTCT is peripartum contamination, rather than in utero exposure. Thus the prophylaxis has a good effectiveness, while such prophylaxis may be less effective in cases of placental transmission, occurring long before delivery.

### 2.3. Effect of Pregnancy on HBV DNA Levels

Even though the immune responses were reported to change during pregnancy, the effect of pregnancy on HBV DNA levels has not been clearly defined. Some studies reported an increasing trend during the course of pregnancy, with a mean of 0.4 log IU/mL to ≥2 log IU/mL [14,15,16]. The results of a study that compared the detection of HBV DNA in asymptomatic HBV carriers between the groups of gestational age of less than 16 weeks, gestational age of 34–36 weeks, and at 6 weeks postpartum reported detection rates of 48%, 59%, and 61%, respectively [17]. However, some studies showed no significant HBV DNA change between trimesters and after delivery [18,19] and between pregnant and non-pregnant women [20]. Hepatitis B e antigen (HBeAg) is generally considered as a marker of HBV replication and infectivity. The patients with HBeAg positivity are usually associated with high serum HBV DNA levels and higher rates of MTCT [21,22]. The serum levels of viral antigens such as HBsAg titers were also stable throughout pregnancy [20]. Some reports suggested that most HBV carriers had stable HBV DNA levels during pregnancy, but increased levels could be found in 5–13% of cases [16,18,19]. However, whether the detection rate of new cases or the incidence of HBV infection is increased or not is still unclear, though theoretically it may be increased because of immune suppression during pregnancy.

### 2.4. Acute Hepatitis in Pregnancy

Acute hepatitis can occur during any period of pregnancy and postpartum, especially asymptomatic or non-specific clinical presentation such as nausea, vomiting, headache, malaise, etc. Individuals with more severe cases may subsequently develop jaundice or other symptoms of liver failure. HBV, which destroys the liver, does not solely account for the mechanism of acute hepatitis and the cause of liver necrosis, but immunological response also plays a role [23]. The course of acute hepatitis in pregnancy does not differ from that of the general population, as reported in some clinical studies, and it was not associated with higher mortality [24,25]. Additionally, there is no significant difference in the occurrence of fulminant hepatitis between pregnant and non-pregnant patients. Although the clinical recovery of acute hepatitis differs little in pregnant and non-pregnant patients, the HBsAg loss and seroconversion is delayed and lower in pregnant patients. Pregnancy might be a possible risk of chronicity following acute HBV infection [24,25], but further studies are necessary to confirm and to elucidate such a finding. The main goal of the treatment of acute hepatitis is to prevent acute liver failure [26]. According to the EASL guideline [26], management of acute hepatitis during pregnancy is not different from that of the non-pregnant patients. The current management of severe acute hepatitis B in pregnancy, especially pharmacological interventions, is inadequate and still needs more data [27]. Therefore, a case-by-case approach should be adopted. Nevertheless, antiviral therapy is rarely necessary because most patients (>95%) have a full spontaneous recovery [26].

### 2.5. Chronic Hepatitis in Pregnancy

Chronic hepatitis during pregnancy with respect to disease progression is of less concern because of its complicated course of progression and the long period required for it to develop clinical symptoms or complications. Therefore, chronic HBV infection during pregnancy with respect to mother-to-child transmission is the principle concern. However, the phases of chronic HBV infection for each individual should be kept in mind to stay alert for complications. The general course of chronic HBV infection is divided into 3 phases: (1) immune-tolerance phase: high HBV DNA and normal ALT levels, (2) immune-active phase: high HBV DNA and elevated ALT levels, and (3) inactive phase: HBV DNA levels < 2000 IU/mL and normal ALT levels. All affected pregnancies should be assessed to obtain the status and phase of infection, and progression to other phases should be closely monitored, which can be both forward and backward progression. The AASLD suggests antiviral therapy to reduce the risk of perinatal transmission of hepatitis B in HBsAg-positive pregnant women with an HBV DNA level >200,000 IU/mL [28]. Antiviral therapy was started at 28–32 weeks of gestation in most of the studies. Antiviral therapy was discontinued at birth to 3 months postpartum in most of the studies. With discontinuation of treatment, women should be monitored for ALT flares every 3 months for 6 months.

Hepatocellular carcinoma (HCC) and cirrhosis are the main end outcomes of chronic HBV infection. Several factors are reported to influence the development of HCC and cirrhosis, including family history of HCC, HBV DNA level above 2000 IU/mL in persons older than 40 years, HBV genotypes C and F, basal core promoter mutation, aflatoxin exposure, and heavy alcohol and tobacco use [29]. Identification of high risk patients is needed to consider early intervention during pregnancy and continuation of intensive postpartum treatment.

### 2.6. Cirrhosis in Pregnancy

Cirrhosis rarely occurs during pregnancy because most cases of cirrhosis occur beyond women’s reproductive age [30]. Another reason is that cirrhosis may cause hypothalamic-pituitary dysfunction, and then anovulation and amenorrhea [31] would occur. Nevertheless, cirrhosis without portal hypertension might not impact sex hormones or fertility. Importantly, however, severe cirrhotic complications that lead to death are more prevalent in pregnant women with cirrhosis. Variceal hemorrhage during vaginal delivery was the most common reason. Mortality rates were significantly different between pregnant women with cirrhosis (7.8%), pregnant women without cirrhosis (0.2%), and non-pregnant women with cirrhosis (2.5%) [32]. Liver transplantation was reported to be successful during pregnancy in cases of decompensation of the liver [33,34], and thus might theoretically be an option in cases of cirrhosis, though more evidence is needed to support.

### 2.7. Hepatitis Flare in Pregnancy

Hepatitis flare or ALT flare can occur during pregnancy and the postpartum period due to the interaction between HBV and the immune response. Because of the suppression of cell-mediated immunity to prevent rejection of the fetus, immune response with high replication of HBV can occur during pregnancy and postpartum periods. Baseline characteristics such as HBV DNA levels, baseline ALT, age, HBeAg status, gravida, and parity were not identified as predictors of hepatitis flares [16].

The definition of hepatitis flare lacks consensus, as shown in Table 1. However, it is consistently accepted that diagnosis of hepatitis flare must include an increase in ALT levels. Different incidences were reported due to different criteria, which may limit the information needed to explain the natural course of hepatitis flare. The risk of hepatitis flare in HBV carriers without antiviral drug was 1.6–14% in the antenatal period and 3.5–50% in the postpartum period [15,16,19,35,36,37,38]; it varies depending on the diagnostic criteria of hepatitis flare. The variations in the reports were further suspected to be a result of an unadjusted normal level of ALT; therefore, the American Association for the Study of Liver Diseases (AASLD) recently defined normal ALT as 35 U/L for males and 25 U/L for females [39]. The rate of hepatitis flare that occurred during the postpartum period was significantly greater than that during pregnancy in both groups of HBV-positive pregnant women with and without antiviral drug. Postpartum hepatitis flare was at its peak at 6 weeks, and 96% would occur within 24 weeks postpartum. Additionally, there was an increased risk of postpartum flare after tenofovir disoproxil fumarate (TDF) treatment withdrawal (45% TDF withdrawal vs. 30% without TDF exposure) [40,41]. TDF, a common drug used to prevent mother-to-child transmission, is considered safe in breastfeeding mothers because only a small amount of TDF is found in breast milk [26,39]. Therefore, some clinicians do not recommend discontinuation of antiviral drug after delivery [42]. However, delayed discontinuation of antiviral drug after a few months of delivery may not reduce the risk of hepatitis flare [38]. Nevertheless, most standard guidelines recommend discontinuing HBV therapy within three months after delivery [26,28,43,44].

In cases without advanced fibrosis or hepatitis delta (HDV) co-infection, most cases of hepatic flare are asymptomatic or have mild and self-limited symptoms [15]; only a few cases progress to hepatic decompensation or jaundice. However, unrecognized viral reactivation or flare can lead to maternal death [45]. ALT level monitoring is suggested in the first 6 months after delivery or 6 months after discontinuation of antiviral medication that was started for vertical transmission prevention [39].

Many obstetric complications may have clinical manifestations mimicking hepatitis flare, for example, hyperemesis gravidarum, pre-eclampsia, hemolysis elevated liver enzyme, and low platelet (HELLP) syndrome, as well as acute fatty liver of pregnancy. Those pregnancy-associated complications, as well as other liver disorders such as hepatitis E virus infection, drug-induced liver injury, etc. should also be included in differential diagnosis.

## 3. HBeAg Seroconversion during Pregnancy and Postpartum

HBeAg seroconversion occurs under immune pressure; immune reactivation, which may lead to elevated ALT and liver inflammation, results in a change in the stage of chronic infection from immune active phase to inactive phase, which shows clinical remission. Compared with non-pregnant women, HBeAg seroconversion in pregnant women increased from 2.2% to 14.3% [46], and 12.5% of seroconversion was observed at 1 year postpartum [47].

## 4. Pregnancy Outcomes

Other than vertical transmission risk of HBV, pregnancy outcomes among pregnant women with HBV infection are typically very good, though adverse pregnancy outcomes resulting from HBV infection have been reported. Progression of disease in HBV carriers to immune-tolerance phase, immune-active phase, or life-threatening conditions, such as cirrhosis and HCC, can occur in pregnant carriers similar to other population groups. These conditions cause increased risk of maternal and fetal mortality [48]. Many studies have been conducted to evaluate the association between pregnancy outcomes and HBV infection. Preterm labor, gestational diabetes mellitus, antepartum hemorrhage, preeclampsia, stillbirth, and miscarriage are some of the pregnancy outcomes that have been studied, and some reports found associations between these outcomes and HBV infection [49,50]. Additionally, our previous study [51] on pregnancy outcomes among HBV carriers found a significantly higher preterm birth rate and higher gestational diabetes mellitus in HBeAg-positive group. Systematic reviews and meta-analysis have been conducted for each adverse pregnancy outcome. For placenta previa and placental abruption, a meta-analysis involving 9088 placenta previa cases and 15,571 placental abruption cases found no association with HBV infection; the odds ratio was 0.98 (95% CI 0.61–1.62) for placenta previa and 1.42 (95% CI 0.93–2.15) for placental abruption [52]. A meta-analysis regarding the association between HBV and gestational diabetes mellitus, involving 439,514 cases, found that HBV infection did not increase the risk of gestational diabetes mellitus; the odds ratio was 1.11 (95% CI 0.96–1.28), but it increased in some special groups (e.g., women from Iran) [53]. For preterm labor, a meta-analysis involving 6781 preterm labor cases found no association with HBV infection; the odds ratio was 1.12 (95% CI 0.94–1.33) [54]. Surprisingly, a meta-analysis involving 11,566 cases evaluated the association between HBV infection and preeclampsia and found a negative association or protective effect of HBV infection on preeclampsia; the odds ratio was 0.77 (95% CI 0.65–0.90) [55]. These systematic reviews and meta-analyses showed that HBV infection had no effect on adverse pregnancy outcomes. Therefore, HBV infection in pregnancy may not need special care regarding this aspect [56]. However, a study later explored the association between HBV infection and preterm labor as well as the main cause of death in children under five years old [57]. The study was a very large population-based cohort study involving 20,827 HBV-positive pregnant women and 489,965 HBV-negative controls. After adjusting for other confounders, the results demonstrated a significant increase in both preterm birth rate before 37 weeks and early preterm birth before 34 weeks of gestation, with 26% higher risk of birth before 37 weeks in HBeAg-negative women (adjusted Relative Risk (aRR) 1.26; 95% CI 1.18–1.34) and 20% in HBeAg-positive women (aRR 1.20; 95% CI 1.08–1.32) as well as 18% higher risk of birth before 34 weeks in HBeAg-negative women (aRR 1.18;95% CI 1.04–1.34) and 34% in HBeAg-positive women (aRR 1.34; 95% CI 1.10–1.61). Finally, they suggested that early detection and proper management would help to improve maternal and neonatal outcomes [58,59]. Lastly, the association between HBV and brachial plexus injury has not been as extensively reported as the other adverse outcomes, but a large population-based study was conducted in the United States. The result of this study demonstrated higher risk of brachial plexus injury in infants born to HBV-positive mothers even after adjusting for the confounding factors (OR 2.04; 95% CI 1.15–3.60) [60].

Zhang et al. [61] conducted a large retrospective cohort study (*n* = 85,190) and showed that pregnant HBV carriers were more likely to present intrahepatic cholestasis of pregnancy (ICP) (OR 3.4, 95% CI 2.80 to 4.13). Additionally, Jiang et al. [62] performed a systematic review and meta-analysis on the association between HBV infection and intrahepatic cholestasis (ICP) and demonstrated a higher risk of ICP with OR of 1.68 (95% CI 1.43–1.97; I2 = 0%) among HBV-positive mothers. Moreover, the mothers with ICP also had increased risk of HBV infection. They also suggest that pregnant women with ICP be screened for HBV infection.

However, the mechanisms causing these outcomes have not been extensively evaluated. Some suspected mechanisms were proposed, such as placental inflammation causing placental abruption [52], the role of HBV in induction of insulin resistance resulting in gestational diabetes mellitus [53], and increased immunotolerance or impaired immune function by HBV resulting in a protective effect for preeclampsia [55]. The possible adverse pregnancy outcomes associated with HBV infection may be summarized as presented in Table 2.

## 5. Mother-to-Child Transmission (MTCT)

Mother-to-child transmission (MTCT) is one of the largest concerns among pregnancies with HBV infection. Acute HBV occurring early in the pregnancy has been associated with a 10% perinatal transmission rate [24]. Transmission rates significantly increase if acute infection occurs at or near the time of delivery, with rates as high as 60% reported [63]. The risk of MTCT of chronic carriers of HBV (the HBsAg-positive mothers) to their babies has been estimated to be as high as 90% in cases of no immunoprophylaxis in the newborns [64]. The transmission can occur in utero, during labor and delivery, and after birth. However, the risk of MTCT can be significantly reduced by immunoprophylaxis (HBV vaccine and hepatitis B immune globulin: HBIG) of all newborns of pregnant women with HBsAg-positivity. As an example, with prophylaxis (HBV vaccine and HBIG as soon as possible after birth, and completed ≥3 dose vaccine series), MTCT was found in only 1.1% of nearly ten thousand infants born to pregnant women with HBsAg-positivity [65].

The immunoprophylaxis shortly after birth with an HBV vaccine together with HBIG is very effective in prevention of MTCT. Nevertheless, up to 25–30% of neonates are still infected with HBV because of immunoprophylaxis failure [66]. The failure rate is likely associated with intrauterine infections. The growing evidence demonstrates that MTCT is significantly associated with positivity of hepatitis B e antigen (HBeAg) and HBV viral load >2000 international units/mL, as well as with age of less than 25 years. Most cases of MTCT occur at the time of delivery, when mucosal membranes of newborns are contaminated with maternal blood and secretions in the birth passage [67]. The most important risk factors for MTCT, in spite of proper prophylaxis (vaccine plus HBIG) are positivity of HBeAg and a high HBV DNA level in the mother. In one classic study, MTCT occurred in the absence of immunoprophylaxis in 85–90% of infants born to the mother with HBeAg-positivity but occurred in 32% of those born to HBeAg-negative mothers [68]. More importantly, infants born to the mothers with HBeAg-positivity, in spite of receiving proper immunoprophylaxis, still had MTCT in approximately 9% of a large cohort study [69]. Additionally, maternal serum HBV DNA levels or viral loads positively correlate with the risk of MTCT. It has been demonstrated that rates of MTCT are found to be 9–39% of infants born to the mothers with high maternal HBV DNA levels, in spite of proper immunoprophylaxis [70,71,72], whereas the risk is rare if maternal HBV DNA level is <10^5^ to 10^6^ international units/mL.

Transplacental transmission may also play a role in fetal HBV infections. We have demonstrated a significant association between maternal levels of viral replication and placental and fetal infection, suggesting that transplacental infection prior to birth may be a mechanism contributing to the higher rates of newborn prophylaxis failure in women with a high viral load [73]. Likewise, Zhang et al. [74] demonstrated that HBV is able to transmit from the mother through the placenta to be confined in the fetal trophoblast. In addition, HBV has been identified in the endothelial cells of villous vessels and the trophoblasts [67,75], suggesting a possible mechanism for intrauterine HBV infection associated with placental barrier breakage. As an example, transplacental transmission can occur because of placental leakage, as seen in cases of a threatened abortion [76]. Placental leakage, commonly seen in preterm labor or abortion, facilitates the disruption of placental barriers and the mixing of maternal and fetal blood, leading to HBV placental transmission [77].

In prevention of MTCT, in addition to immunoprophylaxis of newborns, antiviral therapy for the mothers with high viral loads can significantly reduce the risk of MTCT. Several clinical trials and prospective studies support the use of maternal antiviral therapy to reduce MTCT in the mothers at higher risk [41,78,79], though in a setting with the low rate of MTCT by immunoprophylaxis, the additional maternal use of TDF might not result in a significantly lower rate of transmission [40]. As a consequence, antiviral therapy in pregnant women becomes standard treatment in specifically selected women and is recommended by several organizations, as presented in Table 3. Regarding antiviral therapy, the first-line drug, which is most commonly recommended, is TDF (tenofovir). Women who become pregnant while taking TBV (telbivudine), LAM (lamivudine), or TDF should continue with such medications. However, if they receive other antiviral drugs, switching to TDF is recommended. According to various guidelines, all recommend antiviral therapy with tenofovir or telbivudine for pregnant women with high viral loads, starting at 28–32 weeks of gestation [66].

However, though antiviral therapy is now well accepted for the women at high risk of MTCT, safety, timely initial drug administration, and discontinuation need to be elucidated. For example, Bierhoff et al. [80] are conducting a study to evaluate the procedures following early initiation of maternal TDF, before 20 weeks of pregnancy, to determine the effectiveness, safety, and feasibility of this approach in a low-resource setting.

## 6. Conclusions

Immunological changes induced by pregnancy probably modify the natural course of HBV infection and especially tend to increase hepatitis flare. Growing evidence suggests that placental infection together with hormonal changes caused by pregnancy may be associated with adverse outcomes, especially ICP, GDM, preeclampsia, and preterm birth, etc. Regarding MTCT, immunoprophylaxis failure seems to be associated with high maternal HBV DNA levels and HBeAg positivity. Together with evidence that placental transmission has been consistently demonstrated, in utero infection or placental transmission may also play a role in immunoprophylaxis failure. Antiviral therapy in case of high HBV DNA levels can reduce vertical transmission. Several lines of evidence suggest the effectiveness of antiviral therapy in women with chronic HBV infection with high viral load in addition to hepatitis B immunoglobulin and vaccination for infants.

## Figures and Tables

**Table 1:** Diagnostic criteria and incidence of hepatitis flare from various studies.

Study	Criteria	Incidence
Ter Borg et al. 2008 [30]	↑ ALT level to at least 3 times the baseline	45% during the postpartum period
Nguyen et al. 2014 [31]	↑ ALT level to at least 5 times the upper normal limit	50%: early cessation of antiviral drug; 40%: late cessation of antiviral drug; 29%: untreated during the postpartum period
Giles et al. 2015 [16]	↑ ALT to 2 times the upper normal limit	25% during the postpartum period
Chang et al. 2016 [13]	↑ HBV DNA level to at least 2 log IU/mL *AND* ↑ ALT level to 5 times upper normal limit *OR* 3 times the baseline	6% during pregnancy; 10% during the postpartum period
Kushner et al. 2017 [12]	↑ ALT to 2 times the upper normal limit	14% during pregnancy; 16% during the postpartum period
Liu et al. 2018 [28]	↑ ALT to 2 times the upper normal limit	11.8% in the first trimester; 2.1% at delivery; 9.8% at 1 month postpartum

ALT: Alanine aminotransferase; HBV: Hepatitis B virus; ↑, increased.

**Table 2:** Possible adverse pregnancy outcomes associated with HBV infection.

Adverse Effect	Relative Risk *	Strength of Evidence	Possible Mechanism	Studies (Cohort/Meta-Analysis#)
Miscarriage	↑	Fair	Placental inflammation	Cui et al. 2016 [50]
Preterm birth	↑↑ (in case of HBeAg+)	Fair	Placental inflammation	Tse et al. 2005 [49]; Cui et al. 2016 [50]; Sirilert et al. 2014 [51]; Huang et al. 2014 [54] **#**; Liu et al. 2017 [58]
Gestational diabetes	↑ (in case of HBeAg+)	Weak	Induction of insulin resistance	Tse et al. 2005 [49]; Sirilert et al. 2014 [51]; Kong et al. 2014 [53] **#**
Preeclampsia	↓	Fair	Increased immune tolerance by HBV	Huang et al. 2016 [55] **#**, Zhang et al. 2020 [61]
Placental abruption	↑	Weak	Placental inflammation	Huang et al. 2014 [52] **#**
Fetal growth restriction	No change	Strong	-	Sirilert et al. 2014 [51] Cui et al. 2016 [50]
Intrahepatic cholestasis	↑↑	Strong	Dysregulation of liver function	Zhang et al. 2020 [61], Jiang et al. 2020 [62] **#**

* arbitrary estimation by the authors; ↑ (slightly increased); ↑↑ (obviously increased); ↓ (slightly decreased); # Meta-analysis.

**Table 3:** Some guidelines for prevention of HBV mother-to-child transmission with antiviral therapy (AVT). (This is in addition to the three-dose HBV vaccination, including timely birth dose.)

	Indication	GA for Starting AVT	Discontinuation of AVT	Comments
WHO 2020 [81]	HBV DNA > 200,000 IU/mL	From 28th week of gestation	At least birth	HBeAg testing if HBV DNA not available
RANZ-COG 2019 [82]	HBV DNA levels > 200,000 IU/mL or > 10^6^ copies/mL	Late pregnancy	-	Prefer NIPT more than invasive PND
CDC 2018 [83]	HBV DNA > 200,000 IU/mL	24–28 weeks	Delivery–12 weeks, postpartum	Vaccination in chronic liver disease
EASL 2017 [26]	HBV DNA > 200,000 IU/mL HBsAg > 4 log IU/mL	24–28 weeks	12 weeks, postpartum	
NICE 2017 [84]	HBV DNA > 10^7^ IU/mL	Third trimester	4–12 weeks, postpartum	Monitor HBV DNA every 2 months, ALT monthly
SOGC 2017 [44]	HBV DNA > 200,000 IU/mL	28–32 weeks	Delivery, continuing if high risk of flare	Vaccination in non-immune, invasive PND concern
sMFM 2016 [43]	HBV DNA > 10^6–8^ copies/mL	28–32 weeks	Delivery	High caution for invasive PND
AASLD 2016 [28]	HBV DNA > 200,000 IU/mL	28–32 weeks	Delivery–12 weeks, postpartum	High caution for invasive PND
Thailand * 2015 [85]	HBV DNA > 200,000 IU/mL	24–28 weeks	Delivery–12 weeks postpartum	Vaccination in chronic liver disease
Thailand ** 2018 [86]	Positive HBeAg	28–32 weeks	4 weeks, postpartum	ALT at 6–8 weeks

*/** Representing geographical area of high prevalence (Thailand: Department of Disease Control/Thai Association for the Study of the Liver); AASLD: American Association for the Study of Liver Diseases; sMFM: Society for Maternal-Fetal Medicine; RANZCOG: Royal Australian and New Zealand College of Obstetricians and Gynecologists; EASL: European Association for the Study of the Liver; GA: gestational age; NICE: UK’s National Institute for Health and Care Excellence; NIPT: Non-invasive prenatal test; SOGC: Society of Obstetricians and Gynecologists of Canada; CDC: Centers for Disease Control; WHO: World Health Organization; PND: prenatal diagnosis.

## Data Availability

Not applicable.
